# Clinical characteristics of XP11.2 translocation/TFE3 gene fusion renal cell carcinoma: a systematic review and meta-analysis of observational studies

**DOI:** 10.1186/s12894-016-0154-6

**Published:** 2016-07-11

**Authors:** Xiangming Cheng, Weidong Gan, Gutian Zhang, Xiaogong Li, Hongqian Guo

**Affiliations:** Department of Urology, Nanjing Drum Tower Hospital, the Affiliated Hospital of Nanjing University Medical School, No. 321 Zhongshan Road, Nanjing, 210008 Jiangsu Province China

**Keywords:** Age groups, Carcinoma, Gender identity, Renal cell, Xp11.2 translocation

## Abstract

**Background:**

Renal cell carcinoma (RCC) associated with Xp11.2 translocation/TFE3 gene fusion (Xp11.2 RCC) is a rare subtype of RCC which is firstly described as a distinct entity in 2004 so that clinical characteristics of Xp11.2 RCC in different gender and age are unknown. The purpose of systematic review and meta-analysis is to provide a comprehensive assessment on them.

**Methods:**

MEDLINE, EMBASE and Cochrane databases were searched for studies which evaluate the clinical characteristics of Xp11.2 RCC. The literature published between July 2004 and May 2014 was searched.

**Results:**

A total of 15 studies with 147 participants were included. The meta-analysis demonstrated that number of patients of all age in female was higher than in male with pooled OR of 3.93(95 % CI = 1.66–9.34). However, incidence of distant metastases (OR = 0.34, 95 % CI = 0.12–1.57) and lymphatic metastases (OR = 0.51, 95 % CI = 0.14–1.91), tumor stage (OR = 0.85, 95 % CI = 0.34–2.15) and overall survival (OS) (OR = 0.46, 95 % CI = 0.05–4.34) between male and female were comparable. Incidence in female was higher than in male with pooled OR of 5.13(95 % CI = 1.67–15.72) in adults, while in children no gender-related predominance (OR = 1.19, 95 % CI = 0.38–3.72) was observed. In addition, incidence of distant metastases (OR = 1.00, 95 % CI = 0.13–7.84) and lymphatic metastases (OR = 1.00, 95 % CI = 0.07–13.67) and tumor stage (OR = 1.94, 95 % CI = 0.20–19.03) between children and adults were comparable. Survival curves presented comparable outcomes between male and female (*P =* 0.707) as well as between children and adults (*P =* 0.383).

**Conclusions:**

Female patients with Xp11.2 RCC in adults exhibit a high incidence compared to male, but not in children. Comparable clinical characteristics including incidence of distant and lymphatic metastases, tumor stage and prognosis is presented between male and female as well as between children and adults.

## Background

Renal cell carcinoma (RCC) associated with Xp11.2 translocation/TFE3 gene fusion (Xp11.2 RCC) is a rare subtype of RCC which is delineated as a distinct entity in the 2004 World Health Organization renal tumor classification [1]. This subtype affects primarily children more than adults, accounts for 20–40 % of pediatric RCC and 1–1.6 % of RCC in adults [2]. In addition, Xp11.2 RCC were reported more aggressive than other subtypes of RCC and associated with a poorer prognosis [3].

Xp11.2 RCCs are generally characterized by several translocations on chromosome Xp11.2 resulting in a gene fusion between TFE3 and at least 6 possible partners [4]. Since these translocations are located on the X chromosome, it seems reasonable to suggest that there are gender differences in the clinical characteristics of Xp11.2 RCC. However, comparative analysis regarding the clinical course of Xp11.2 RCC between male and female remains controversial due to relatively rare incidence [5–8]. In addition, as Xp11.2 RCC predominantly among children and associated with a poorer prognosis, the comparison of the prognosis between children and adults exists as well [9]. Due to such controversy in gender and age of Xp11.2 RCC, in this systematic review and meta-analysis, we studied the clinical characteristics of Xp11.2 RCC regarding patient demographics, incidence of distant and lymphatic metastases, tumor stage and prognosis in order to better define the difference of Xp11.2 RCC between male and female as well as between children and adults.

## Methods

### Literature search

The present meta-analysis was conducted following the Preferred Reporting Items for Systematic Reviews and Meta-Analyses (PRISMA) statement (accessible at http://www.prisma-statement.org/). A computer-aided literature search was performed in May 2014 with usage of the Cochrane, MEDLINE, EMBASE and Science Citation Index. An initial search strategy use keywords describing TFE3, Xp11.2 renal cell carcinoma. The literature published between July 2004 and May 2014 was searched since Xp11.2 RCC was first definitely diagnosed as a distinct entity in 2004. Additional studies were searched by reference lists from primary studies to identify any studies missed by the electronic search strategies.

### Study selection

Two authors reviewed abstracts of all candidate articles independently and read full-text review if articles could not be categorized based on title and abstract alone. All articles were checked for unified inclusion criteria. Discrepancies were resolved by consensus with a third author. Authors of included studies were not contacted for additional, unreported data.

### Study inclusion/exclusion criteria

Studies were included if they fulfilled the following criteria: (1) Studies reported clinical parameters of patients with Xp11.2 RCC including gender and age. (2) The diagnosis of all patients with Xp11.2 RCC was confirmed by immunohistochemical (IHC) assay for TFE3 combined with fluorescence in-situ hybridization polyclonal (FISH) assay or other strict criteria. When the results of FISH assay and other molecular biology such as Reverse Transcription - Polymerase Chain Reaction (RT-PCR) were contradicted with IHC assay, definite diagnosis of Xp11.2 RCC was made by genetic analysis including FISH assay and other molecular biology. Inclusion was not limited to randomized controlled trials (RCTs). Studies were excluded if they were (1) performed in the lab with animal or cell models; (2) reviews and case reports with only one case due to one case was not enough to compare the gender and age diference; (3) molecular or mechanism researches. When duplicate patient populations were published from on institution, the most recent data were used.

### Quality assessment of primary

Quality of primary articles was assessed in duplicate by independent authors. Issues such as number of case, manner of diagnosis and study design were analyzed. Any disagreement was dealt with consensus and discussion with a third author.

### Data extraction

Data extraction fields for each study included the following: (1) demographic data concerning patient gender, age, treatment and condition during follow-up period; (2) tumor data for different gender and age including stage, lymphatic and distant metastases. For age, patients was divided into children (≤ 14 years) and adults (> 14 years). When gender-related incidences in children and adults were studied, reference would be excluded if only 1 patient was presented in the study. The cases of Xp11.2 RCC would be excluded if the lymphatic and distant metastases of tumor cannot be determined when we studied tumor metastases and stage. If journal articles contained insufficient information, we attempted to contact authors to obtain missing details. If failed, we could just present out the existing results.

### Data synthesis and analysis

The clinical data included number of Xp11.2 RCC in different gender and age for which tumor stage, lymphatic metastases, distant metastases and prognosis such as overall survival (OS) were analyzed. All above data were recorded from original articles. The statistical software Review Manager (Version 5.0 for Windows) was applied to carry out all the analysis. Data regarding incidence of Xp11.2 RCC, tumor stage, metastases and overall survival were dichotomous data which was shown as odds ratios (ORs). For dichotomous data, when no statistically significant heterogeneity was detected, Mantel-Haenszel fixed-effect model was used to pool ORs with 95 % confidence intervals (CIs). The heterogeneity across the studies was investigated using a χ2-based test of homogeneity and evaluation of the inconsistency index (I^2^) statistic. The p value less than 0.1 was considered as statistically significant heterogeneity. An I^2^ value > 50 % was considered to represent substantial heterogeneity across studies, which was the symbol of applying random- effect model. Publication bias for each of the pooled study groups was assessed by funnel plot if the number of studies in group was ≥ 10.

We also estimated the survival curves in different gender and age, all of whom were treated by surgical procedure such as nephron sparing surgery (NSS) and radical nephrectomy (RN). The probability of survival was analyzed by the Kaplan-Meier methods within the log-rank test. Reported *p* values were statistical significance set at *p <* 0.05.

## Results

### Literature and study characteristics

**Fig. 1:**
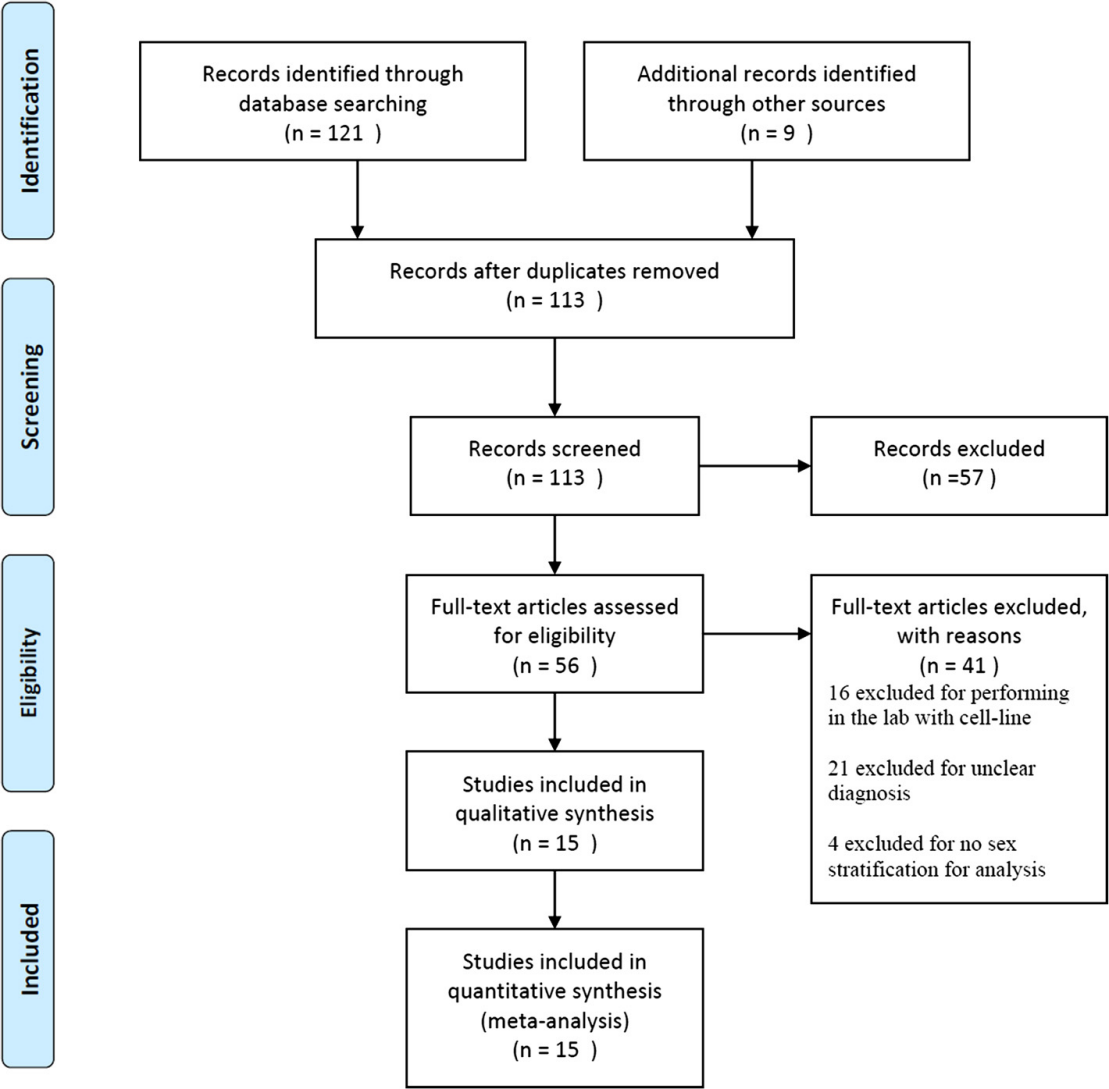
PRISMA Flow Diagram of study selection for Meta- analysis

**Fig. 2:**
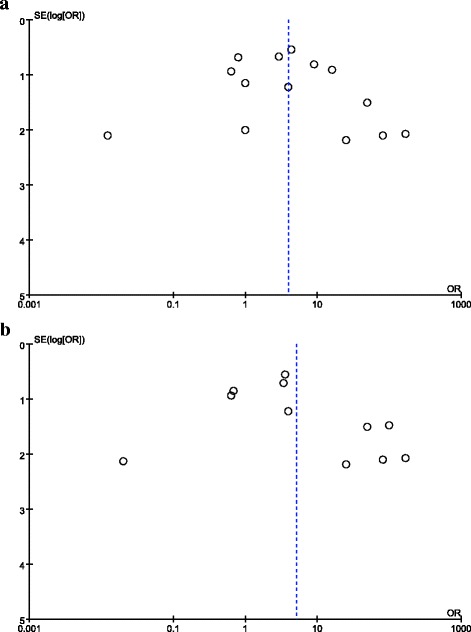
Publication bias assessment for study of distribution of Xp11.2 RCC in male and female. Funnel plots show that there was no evidence for significant publication bias in any of the 2 pooled groups. **a** patients of all ages; **b** patients older than 14

As shown in Fig. 1, 15 studies [4, 10–23] with 147 subjects met the inclusion criteria. All of the enrolled studies were retrospective, in which 2 of them provided all the clinical parameters in different gender while the other gave part of these parameters, as shown in Table 1. None of study provided detailed information about OS in comparison of different age while other clinical parameters were shown in Table 2. The number of studies in the pooled group of gender-related incidence in adults were ≥ 10, as well as patients of all ages, publication bias of the groups were described as visual assessment of a funnel plot in Fig. 2.

**Table 1:** Clinical characteristics of included studies between male and female

Reference	Number of patients		Incidence of lymphatic metastases		Incidence of distant metastases		Stage I/II (III/IV)		Overall survival	
	Male	Female	Male	Female	Male	Female	Male	Female	Male	Female
Rao et al. [20]	9	8	1/3	0/4	1/1	0/1	2/7	4/4	--	--
Hodge et al. [21]	7	12	--	--	--	--	--	--	--	--
Altinok et al. [10]	3	3	2/3	1/3	--	--	1/2	2/3	0/3	0/3
Zou et al. [22]	5	4	2/5	1/4	1/5	0/4	3/2	1/3	3/4	3/4
Hung et al. [13]	1	7	0/1	2/7	0/1	2/7	1/0	3/4	--	--
Green et al. [18]	10	21	0/0	4/5	1/1	3/4	4/6	9/12	--	--
Zhong et al. [16]	0	6	--	--	--	5/6	--	--	--	--
Choueiri et al. [11]	3	12	--	--	--	--	--	--	--	--
Pflueger et al. [19]	4	12	1/2	3/4	1/1	0/3	--	1/3	--	--
Gaillot-Durand et al. [17]	0	2	--	--	--	--	--	--	--	--
Zhong et al. [12]	0	4	--	--	--	--	--	--	--	--
Klatte et al. [4]	1	1	1/1	0/1	1/1	0/1	0/1	1/0	0/1	0/1
Sukov et al. [15]	2	4	--	1/2	0/2	1/4	--	--	2/2	2/4
Kim et al. [14]	4	0	--	--	--	--	--	--	--	--
Argani et al. [23]	0	2	--	--	--	--	--	--	--	--

**Table 2:** Clinical characteristics of included studies between children and adults

Reference	Gender		Incidence of lymphatic metastases		Incidence of distant metastases		Stage I/II (III/IV)	
	Children (Male/Female)	Adults (Male/Female)	Children	Adults	Children	Adults	Children	Adults
Rao et al. [20]	3/3	6/5	0/5	1/2	0/1	1/1	0/1	0/1
Hodge et al. [21]	1/1	6/11	--	--	--	--	--	--
Altinok et al. [10]	3/3	--	--	--	--	--	--	--
Zou et al. [22]	--	5/4	--	--	--	--	--	--
Hung et al. [13]	--	1/7	--	--	--	--	--	--
Green et al. [18]	0/2	10/19	--	4/5	1/1	4/5	1/1	14/14
Zhong et al. [16]	--	0/6	--		--	5/6	--	--
Pflueger et al. [19]	3/2	1/10	1/1	3/5	--	0/2	--	1/1
Gaillot-Durand et al. [17]	--	0/2	--	--	--	--	--	--
Zhong et al. [12]	--	0/4	--	--	--	--	--	--
Klatte et al. [4]	1/0	0/1	1/1	0/1	1/1	0/1	0/1	1/0
Sukov et al. [15]	--	2/4	--	--	--	--	--	--
Kim et al. [14]	1/0	3/0	--	--	--	--	--	--
Argani et al. [23]	--	0/2	--	--	--	--	--	--

### Primary outcomes

**Fig. 3:**
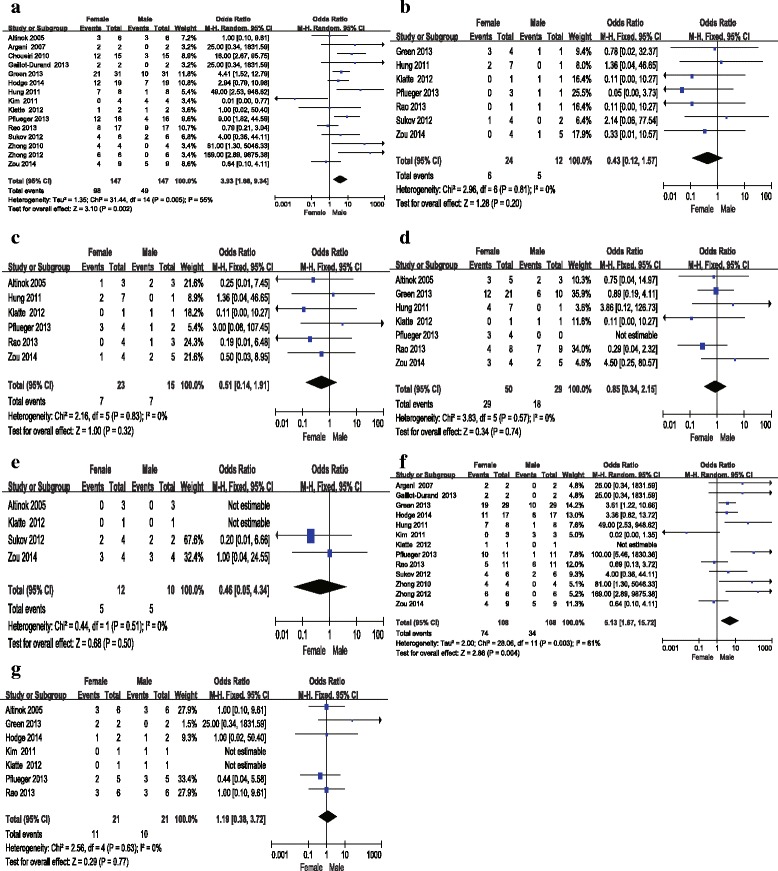
Forrest plots and meta-analysis of studies showing 95 % confidence interval of male Xp11.2 RCC as compared with female Xp11.2 RCC. Clinical characteristics are reported as follows: **a** Number of patients of all ages; **b** Incidence of distant metastases; **c** Incidence of lymphatic metastases; **d** Tumor stage; **e** Overall survival (OS); **f** Different gender-related incidence in adults; **g** Different gender-related incidence in children

**Fig. 4:**
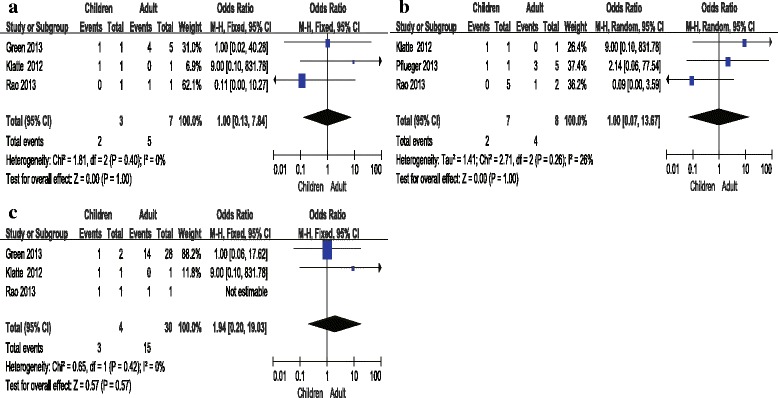
Forrest plots and meta-analysis of studies showing 95 % confidence interval of Xp11.2 RCC in children as compared with adults. Clinical characteristics are reported as follows: **a** Incidence of distant metastases; **b** Incidence of lymphatic metastases; **c** Tumor stage

The forest plots about gender-related differences and age-related differences were shown in Figs. 3 and 4. In study of gender-related difference in incidence of Xp11.2, subgroup analysis was made to learn gender-related incidence in children and adults.

### Gender

**Fig. 5:**
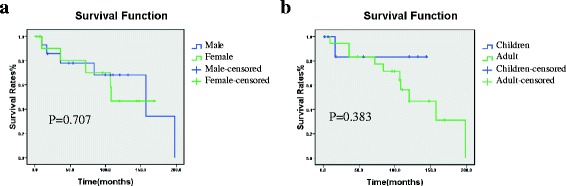
The survival function of patients. **a** Overall survival curve between male and female. **b** Overall survival curve between children and adult

Totally, the number of included patients was 147 (*n =* 98 for women and *n =* 49 for men). We observed that *I*^2^ value was > 50 % in the group of incidence of patients of all age in different gender (*I*^2^ statistic = 55 %, *P =* 0.005). Therefore we applied random- effect model in meta-analysis of this data. Meanwhile, no evidence for significant publication bias was observed in this pooled group. For other group, there was no evidence for heterogeneity about incidence of distant (*I*^2^ statistic = 0 %, *P =* 0.81) and lymphatic metastases *(I*^2^ statistic = 0 %, *P =* 0.83), tumor stage (*I*^2^ statistic = 0 %, *P =* 0.57) and OS (*I*^2^ statistic = 0 %, *P =* 0.51). Results of meta-analysis demonstrated that the incidence of Xp11.2 in female was significantly higher than in male with pooled OR of 3.93(95 % CI = 1.66–9.34). However, incidence of distant metastases (OR = 0.43, 95 % CI = 0.12–1.57) and lymphatic metastases (OR = 0.51, 95 % CI = 0.14–1.91, tumor stage (OR = 0.85, 95 % CI = 0.34–2.15) and overall survival (OS) (OR = 0.46, 95 % CI = 0.05–4.34) between male and female were comparable. The result of OS consistent with analysis for clinical characteristics of Xp11.2 RCC was confirmed by survival function (*P =* 0.707), as shown in Fig. 5a.

### Age

A total of 132 cases of Xp11.2 from 13 studies including 23 children (≤ 14 years) and 109 adults (> 14 years) were pooled for analyzing the clinical characteristics between adults and children. *I*^2^ value was > 50 % in the group of gender-related incidence in adults (*I*^2^ statistic = 61 %, *P =* 0.003) so that random- effect model was applied. According to funnel plot, there was no significant publication bias in this study. *P* value was >  0.10 in group of gender-related incidence in children (*I*^2^ statistic = 0 %, *P =* 0.63) and incidence of distant (*I*^2^ statistic = 0 %, *P =* 0.40) and lymphatic metastases *(I*^2^ statistic = 26 %, *P* = 0.26) between children and adults as well as in tumor stage (*I*^2^ statistic = 0 %, *P* = 0.42). Results of meta-analysis demonstrated that the incidence of patients in female was significantly higher than in male with pooled OR of 5.13(95 % CI = 1.67–15.72) in adults while in children no gender-related predominance (OR = 1.19, 95 % CI = 0.38–3.72) was observed. In addition, incidence of distant metastases (OR = 1.00, 95 % CI = 0.13–7.84) and lymphatic metastases (OR = 1.00, 95 % CI = 0.07–13.67) and tumor stage (OR = 1.94, 95 % CI = 0.20–19.03) between children and adults were comparable. The result of clinical characteristics of Xp11.2 RCC was confirmed by survival function (*P =* 0.383), as shown in Fig. 5b.

## Discussion

Xp11.2 RCC is a rare subtype of RCC which results from gene fusions between the transcription factor E3 (TFE3) gene and at least 5 fusion partners including ASPL-TFE3, PRCC-TFE3, PSF-TFE3, CLTC-TFE3, and Nono -TFE3, whose chromosomal rearrangement is t(X;17)(p11.2;q25), t(X;1)(p11.2;q21), t(X;1)(p11.2;p34), t(X;17)(p11.2;q23) and inv(X)(p11.2;q12), respectively [8, 24]. Due to the translocations lead to overexpression of TFE3 protein, detection of TFE3 protein by IHC assay is currently the most commonly used diagnostic technique in clinical practice [25]. Gaillot-Durand et al. showed that nuclei stained with an intensity of ++ to +++ in IHC assay was necessary to suspect the diagnosis of Xp11.2 RCC [17]. However, recent studies have found that the positive predictive value of positive TFE3 staining for Xp11.2 RCC is very low as well as highly false positive results [4, 14, 17, 18]. Definite diagnosis of Xp11.2 RCC should be not only made by IHC assay but also by such strict criteria as FISH assay and other molecular biology [4, 17]. Thus, the strength of this study is that we first analyzed the clinical characteristics of Xp11.2 RCC with the diagnostic criteria of the combined examination of TFE3 IHC staining and other molecular biology. In our report, 21 studies were excluded from meta-analysis because they made the diagnosis of only by IHC assay, which may be suspected to have other subtypes of RCC included.

Gender-related difference in incidence of Xp11.2 RCC remains controversial. Several previous studies indicated a female predominance in incidence of Xp11.2 RCC [7, 8, 26], while a few reports showed a male predominance in Xp11.2 RCC [5, 22]. In addition, Altinok et al. found the male/female ratio was equal in their study of pediatric Xp11.2 RCC [10]. We gathered all references which met the inclusion criteria for meta-analysis and found that the number of patients of all ages in female was significantly higher than that in male with pooled OR of 3.93 (95 % CI = 1.66–9.34). To learn gender-related incidence of Xp11.2 RCC more comprehensively, included patients were divided into two groups as children (≤14 years, *n =* 23) and adults (>14 years, *n =* 109). We found that female predominance is seen in adults (OR = 5.13, 95 % CI = 1.67–15.72) and no gender difference in children (OR = 1.19, 95 % CI = 0.38–3.72). Although incidence of Xp11.2 RCC in children is higher than in adults as a percentage of RCC [2], adult Xp11.2 RCC could still outnumber pediatric Xp11.2 RCC due to RCC is more common in adults (approximately 25,000 cases per year in the United States) than in children (approximately 25 cases per year in the United States) [23]. As previously mentioned, translocations are mainly located on the X chromosome. Moreover, translocations might only occur on the active X chromosome but not the Barr body (inactive X chromosome) although females have two X chromosomes. According to Sirchia [27], homozygosity (two active X chromosome) can exist in normal somatic cell due to mutations occur during mitosis. Thus, the possible explanation for gender-related difference existing in adults but not in children is that a higher level of homozygosity in adults than children results from accumulation of mutations with age. Nonetheless, more comprehensive and detailed studies are required to confirm this speculation.

At diagnosis, metastases of Xp11.2 may occur in about one-third of patients [28], who were older and predominant in male [6]. Moreover, some studies showed that Xp11.2 RCC has a higher degree of invasiveness and a more rapid disease course in adult patients than children [29, 30]. Nevertheless, a study reporting contradictory data to that showed a case of children with bone metastases at diagnosis and a very aggressive course [28]. In our meta-analysis, the incidence of distant and lymphatic metastases and tumor stage between male and female were comparable as well as between children and adults. The important reason for the inconsistencies may be explained by the differences of diagnostic methods. Previously mentioned diagnosis was only based on IHC assay, while this study adopted the combined TFE3 IHC staining and FISH assay to exclude the possibility of other subtypes of RCC.

The treatment for Xp11.2 RCC is still not well defined. For Xp11.2 RCC including that with positive regional lymph nodes, surgery is the optional treatment [26] while NSS is an alternative treatment for patients with tumors measuring < 7 cm [29]. Chemotherapy such as sunitinib can be applied as well [31]. However, Xp11.2 has poorer prognosis no matter what treatment is applied [4, 32]. Recent studies reported that prognosis of pediatric Xp11.2 RCC was better than that of adult Xp11.2 RCC [30, 33, 34]. However, such conclusion was based on small sample size without strict diagnosis. In our meta-analysis, we grouped patients who have been treated by RN or NSS to analyze the prognosis of RCC. No statistically significant difference was observed in the probability of survival between children and adults. In addition, we analyzed prognosis in male and female by survival curve and OS which showed no significant difference. Thus, this meta-analysis suggested that the prognosis of Xp11.2 RCC between male and female is comparable as well as between children and adults.

There are some limitations in this meta-analysis. The sample size was still small and most of studies cannot provide completely detailed information. In addition, the number of existing high-quality studies is too small to compare parameters about prognosis such as free progress survival. Further studies are required to solve these problems.

## Conclusion

This meta-analysis of the current evidence illustrate that the female patients with Xp11.2 RCC in adults exhibit a high incidence compared to the male, but not in children. Comparable clinical characteristics including incidence of distant and lymphatic metastases, tumor stage and prognosis is presented between male and female as well as between children and adults. Based on these results, the epidemiological information about gender difference in incidence of Xp11.2 RCC in adults is provided. Further investigations on more comprehensive and heterogeneous studies should be carried out to extend our results.

## Abbreviations

RCC, Renal cell carcinoma; Xp11.2 RCC, Renal cell carcinoma associated with Xp11.2 translocation/TFE3 gene fusion; OS, Overall survival; IHC, immunohistochemical; FISH, fluorescence in-situ hybridization polyclonal; RT-PCR, Reverse Transcription - Polymerase Chain Reaction; RCTs, randomized controlled rials; NSS, nephron sparing surgery; RN; radical nephrectomy; CIs, confidence intervals

## References

[1] Bruder E, Passera O, Harms D (2004). Morphologic and molecular characterization of renal cell carcinoma in children and young adults. Am J Surg Pathol.

[2] Kmetec A, Jeruc J (2014). Xp 11.2 translocation renal carcinoma in young adults; recently classified distinct subtype. Radiol Oncol.

[3] Rao Q, Guan B, Zhou XJ (2010). Xp11.2 Translocation Carcinomas Have a Poorer Prognosis Than Non-Xp11.2 Translocation Carcinomas in Children and Young Adults: A Meta-Analysis. Int J Surg Pathol.

[4] Klatte T, Streubel B, Wrba F (2012). Renal cell carcinoma associated with transcription factor E3 expression and Xp11.2 translocation: incidence, characteristics, and prognosis. Am J Clin Pathol.

[5] Wu A, Kunju LP, Cheng L, Shah RB (2008). Renal cell carcinoma in children and young adults: analysis of clinicopathological, immunohistochemical and molecular characteristics with an emphasis on the spectrum of Xp11.2 translocation-associated and unusual clear cell subtypes. Histopathology.

[6] Malouf GG, Camparo P, Molinie V (2011). Transcription factor E3 and transcription factor EB renal cell carcinomas: clinical features, biological behavior and prognostic factors. J Urol.

[7] Su HH, Sung MT, Chiang PH, Cheng YT, Chen YT (2014). The preliminary experiences of translocation renal cell carcinoma and literature review. Kaohsiung J Med Sci.

[8] Wang W, Ding J, Li Y (2014). Magnetic Resonance Imaging and Computed Tomography Characteristics of Renal Cell Carcinoma Associated with Xp11.2 Translocation/TFE3 Gene Fusion. PLoS One.

[9] Spreafico F, Collini P, Terenziani M, Marchiano A, Piva L (2010). Renal cell carcinoma in children and adolescents. Expert Rev Anticancer Ther.

[10] Altinok G, Kattar MM, Mohamed A, Poulik J, Grignon D, Rabah R (2005). Pediatric renal carcinoma associated with Xp11.2 translocations/TFE3 gene fusions and clinicopathologic associations. Pediatr Dev Pathol.

[11] Choueiri TK, Lim ZD, Hirsch MS (2010). Vascular endothelial growth factor-targeted therapy for the treatment of adult metastatic Xp11.2 translocation renal cell carcinoma. Cancer.

[12] Zhong M, De Angelo P, Osborne L (2010). Dual-color, break-apart FISH assay on paraffin-embedded tissues as an adjunct to diagnosis of Xp11 translocation renal cell carcinoma and alveolar soft part sarcoma. Am J Surg Pathol.

[13] Hung CC, Pan CC, Lin CC, Lin AT, Chen KK, Chang YH (2011). XP11.2 translocation renal cell carcinoma: clinical experience of Taipei Veterans General Hospital. J Chin Med Assoc.

[14] Kim SH, Choi Y, Jeong HY, Lee K, Chae JY, Moon KC (2011). Usefulness of a break-apart FISH assay in the diagnosis of Xp11.2 translocation renal cell carcinoma. Virchows Arch.

[15] Sukov WR, Hodge JC, Lohse CM (2012). TFE3 rearrangements in adult renal cell carcinoma: clinical and pathologic features with outcome in a large series of consecutively treated patients. Am J Surg Pathol.

[16] Zhong M, De Angelo P, Osborne L (2012). Translocation renal cell carcinomas in adults: a single-institution experience. Am J Surg Pathol.

[17] Gaillot-Durand L, Chevallier M, Colombel M (2013). Diagnosis of Xp11 translocation renal cell carcinomas in adult patients under 50 years: interest and pitfalls of automated immunohistochemical detection of TFE3 protein. Pathol Res Pract.

[18] Green WM, Yonescu R, Morsberger L (2013). Utilization of a TFE3 break-apart FISH assay in a renal tumor consultation service. Am J Surg Pathol.

[19] Pflueger D, Sboner A, Storz M (2013). Identification of molecular tumor markers in renal cell carcinomas with TFE3 protein expression by RNA sequencing. Neoplasia.

[20] Rao Q, Williamson SR, Zhang S (2013). TFE3 break-apart FISH has a higher sensitivity for Xp11.2 translocation-associated renal cell carcinoma compared with TFE3 or cathepsin K immunohistochemical staining alone: expanding the morphologic spectrum. Am J Surg Pathol.

[21] Hodge JC, Pearce KE, Wang X, Wiktor AE, Oliveira AM, Greipp PT (2014). Molecular cytogenetic analysis for TFE3 rearrangement in Xp11.2 renal cell carcinoma and alveolar soft part sarcoma: validation and clinical experience with 75 cases. Mod Pathol.

[22] Zou H, Kang X, Pang LJ (2014). Xp11 translocation renal cell carcinoma in adults: a clinicopathological and comparative genomic hybridization study. Int J Clin Exp Pathol.

[23] Argani P, Olgac S, Tickoo SK (2007). Xp11 translocation renal cell carcinoma in adults: expanded clinical, pathologic, and genetic spectrum. Am J Surg Pathol.

[24] Liu K, Xie P, Peng W, Zhou Z (2014). Renal carcinomas associated with Xp11.2 translocations/TFE3 gene fusions: findings on MRI and computed tomography imaging. J Magn Reson Imaging.

[25] Komai Y, Fujiwara M, Fujii Y (2009). Adult Xp11 translocation renal cell carcinoma diagnosed by cytogenetics and immunohistochemistry. Clin Cancer Res.

[26] Ahluwalia P, Nair B, Kumar G (2013). Renal Cell Carcinoma Associated with Xp11.2 Translocation/TFE3 Gene Fusion: A Rare Case Report with Review of the Literature. Case Reports Urology.

[27] Sirchia SM, Ramoscelli L, Grati FR (2005). Loss of the inactive X chromosome and replication of the active X in BRCA1-defective and wild-type breast cancer cells. Cancer Res.

[28] Sudour-Bonnange H, Leroy X, Chauvet M, Classe M, Robin PM, Leblond P (2014). Cutaneous metastases during an aggressive course of Xp11.2 translocation renal cell carcinoma in a teenager. Pediatr Blood Cancer.

[29] Song HC, Sun N, Zhang WP, He L, Fu L, Huang C (2014). Biological characteristics of pediatric renal cell carcinoma associated with Xp11.2 translocations/TFE3 gene fusions. J Pediatr Surg.

[30] Armah HB, Parwani AV (2010). Xp11.2 translocation renal cell carcinoma. Arch Pathol Lab Med.

[31] Numakura K, Tsuchiya N, Yuasa T (2011). A case study of metastatic Xp11.2 translocation renal cell carcinoma effectively treated with sunitinib. Int J Clin Oncol.

[32] Qiu R, Bing G, Zhou XJ (2010). Xp11.2 Translocation renal cell carcinomas have a poorer prognosis than non-Xp11.2 translocation carcinomas in children and young adults: a meta-analysis. Int J Surg Pathol.

[33] Meyer PN, Clark JI, Flanigan RC, Picken MM (2007). Xp11.2 translocation renal cell carcinoma with very aggressive course in five adults. Am J Clin Pathol.

[34] Koie T, Yoneyama T, Hashimoto Y (2009). An aggressive course of Xp11 translocation renal cell carcinoma in a 28-year-old man. Int J Urol.

